# The Shopping Behavior of International Students in Poland during COVID-19 Pandemic

**DOI:** 10.3390/ijerph191811311

**Published:** 2022-09-08

**Authors:** Julita Szlachciuk, Olena Kulykovets, Maciej Dębski, Adriana Krawczyk, Hanna Górska-Warsewicz

**Affiliations:** 1 Department of Food Market and Consumer Research, Institute of Human Nutrition Sciences, Warsaw University of Life Sciences, 02-787 Warsaw, Poland; 2 Department of Marketing and Tourism, Faculty of Management and Security Sciences, University of Social Sciences, 00-635 Warsaw, Poland; 3 Centre for Applied Research on Education, Amsterdam School of International Business, 1102 CV Amsterdam, The Netherlands

**Keywords:** COVID-19, consumer behavior, shopping behavior, international students, food, consumer

## Abstract

The purpose of this research is to analyze the shopping behavior of international students (Asian vs. European) studying in Poland. Participants were recruited from universities located in Warsaw between June and September 2020. A total of 806 questionnaires were collected, 87 of which were eliminated due to non-response. The research sample consisted of 719 people. We conducted an exploratory factor analysis and cluster analysis for the entire study population and separately for European and Asian students. In exploratory factor analysis, two factors were extracted for the entire population, while three factors each were extracted for the European and Asian student groups. In cluster analysis, we obtained four clusters each for the entire study population and the group of European and Asian students. Our study found that among Asian students, compared to European students, there was a greater change in shopping behavior during the COVID-19 pandemic, expressed by a greater preference for online shopping, greater purchases of fruits and vegetables, purchases of local products, and shorter shopping time.

## 1. Introduction

One of the basic elements of consumer behavior is making purchasing choices. Diverse households have different approaches to the purchasing process. For some, it is a recreational element, while for others, it is a challenge comparable to a gainful activity [1]. Shopping behaviors can be referred to as the category of specific behaviors that primarily reflect human needs and desires, as well as material and spiritual interests [2]. Changes in purchasing behavior may occur under the influence of social, demographic, cultural, and situational factors [3].

Culture is one of the elements that influence consumers’ purchase choices [4], sensory preferences, or attitudes towards novel foods [5]. According to Doole and Lowe, culture is something people learn as they grow up in their immediate environment [6]. The consumers’ cultural background influences the perception of food, and this, in turn, affects the amount of consumption and the acceptability of the food [7]. Cultural factors appear to have a major influence on food choice behavior than the genetic variation between subjects in cross-cultural research [8]. The ability to anticipate and maximize consumer preferences requires a deep understanding of cultural aspects [9]. Culture plays a very important role in influencing consumer behaviors [10]. Culture can influence the thoughts and behavior of consumers. Moreover, it has a different influence on the functioning of various institutions, including the media. Consumers from different cultural groups have different ways of consumption due to, at least, differences in norms and values [11].

Some researchers agree that a developed habit can influence the attitude towards online shopping [12]. Demographics, lifestyle, and cultural variables also influence online shopping habits. Particularly large differences can be found between the purchasing behavior of consumers in developed and developing countries [13]. One study showed a significant influence of culture on the adaptation of information technology, as well as the role of cultural differences in the implementation and acceptance of the e-commerce market by consumers [14].

When faced with making a choice, the consumer may be influenced by both internal stimuli (such as, for example, prior knowledge of the product or brand) and external discernment, which consists of searching for more information, recommendations, and opinions of others about the product or brand [15]. One of the most interesting things in the process of making consumer decisions is the so-called flexibility. The consumer, while remaining in a changing environment, can make various choices under the influence of both external and internal factors. Thus, consumers vary in their choice of strategy when making purchasing decisions [16]. Consumers also have different attitudes to shopping, just as their thoughts, preferences, and emotions are different [17].

Consumers’ choice of where to buy reflects their perception of distance, price, the time needed to make the planned purchases, and the degree of enjoyment in making the purchasing activity [18], which allowed it to penetrate almost all industries [19]. The dynamic development of search engines through the creation of the so-called intelligent search, as well as the convenience of remote shopping with the ability to place an order immediately, have a huge impact on the development of online shopping [20].

Online shopping aligns with modern consumers’ demand for fast and efficient consumption [21]. Although the experience and appearance remain the advantage of traditional retail stores over online stores, they are also important factors that can influence consumer decisions [22]. Research from previous years showed that in the process of making a product selection, the type of product influences the type of information retrieval, which ultimately also affects the consumer’s decision-making process [23,24,25].

Traditional and online consumer purchasing channels also differ significantly in the level of effort that consumers must make and the level of uncertainty that accompanies each step of the decision-making process [26]. The attitude towards online shopping and the intention to buy online are influenced not only by the ease of use, pleasure, and usability but also by the characteristics of the consumer himself, product features, situational factors, trust in online shopping, and previous experiences with online shopping [27]. Online shopping satisfaction may also be influenced by factors such as the choice of product preferences, a wider range, and availability of products, as well as available service after the end of the product sale process [28], the security and convenience of concluded financial transactions, the appearance of the website [29], convenience and merchandising (assortment and availability of product information) [30].

According to some researchers, perceived risk is one of the basic elements that may influence the choice of where to buy, especially in the context of online shopping [31,32]. The influence of risk perception is a phenomenon that occurs both in social sciences [33,34] and economics [35]. The crisis related to the spread of the SARS-CoV-2 (COVID-19) virus poses the same risk associated with hazardous conditions for human health and life as in the case of a disaster and can be considered an anthropogenic crisis [36].

Due to the COVID-19 pandemic, the e-commerce industry faced new challenges related to a deficit in the retail system [37] and logistics, especially when it comes to the supply chain between the broker and end recipients [38,39]. Similar consumer behavior during the crisis was observed in both developed economies and developing countries [40].

The COVID-19 pandemic can be identified as a crisis that changed consumer behavior as well as affected logistics in the final supply chain [37]. Moreover, initially, the COVID-19 pandemic had a significant negative impact on the consumption process of households worldwide [41,42]. Online shopping has increased significantly, and food consumption patterns have changed rapidly [43].

Researchers Baarsma B. and Groenewegen J. noticed that more words related to the COVID-19 pandemic cause a decline in the variety of products purchased, indicating a tendency to buy more of the same, which may indicate hoarding behavior [44]. In another study, authors Chang and Meyerhoefer found that each additional confirmed COVID-19 case increased the number of online shoppers shopping for groceries by 4.9% and sales by 5.7% [45].

Changes in consumer behavior caused by the COVID-19 pandemic are fast, dynamic, and unpredictable [46]. COVID-19 has a significant impact on the culture and economy around the world, which will consequently affect the daily lives of consumers, as well as the way they interact with the places where products and services are sold [47]. Researchers from Grashuis, Skevas, and Segovia have indicated that there has been a significant upward shift in sales of groceries purchased online from 4% in the pre-pandemic period to almost 15% in the pandemic period [48]. The reason for such a large increase in online shopping interest may be a rapid transformation driven by increased consumer interest [49].

Consumers now have higher expectations regarding the safety of purchases in brick-and-mortar stores, which results in a decrease in the frequency and duration of offline purchases and an increase in online purchases [2]. Interestingly, fear caused by the COVID-19 pandemic among consumers contributed to an increase in impulsive behavior when making purchasing decisions [50,51]. Consumers now also prefer packaged products and try to avoid un-packaged, shop faster and more efficiently, using more digital payments and online purchases [52].

The COVID-19 pandemic has had an impact on consumer shopping behavior. Consumers in an environment where COVID-19 spreads faster prefer to purchase remotely. On the other hand, where COVID-19 spreads more slowly, the need to make purchases remotely is much lower, as well as the demand for the service of delivering purchases to the consumer. Moreover, in addition to the price and method of product delivery, the time criterion for delivering products to the consumer is also becoming important [48]. During the pandemic, consumers began to buy more staple foods with a longer shelf life, as well as household and cleaning products and frozen food [53]. The increased impact of social media, social distancing, and a general lockdown have also led to a shopping panic that has become a global phenomenon. Impulsiveness when shopping also led to a shortage of groceries, household products, cosmetics, and some personal protective equipment on store shelves [54,55].

Fanelli’s study classified the impact of the pandemic into three categories: changes in food purchases, food patterns, and food habits [56]. The results of qualitative research by Filimonau et al. showed great consumer interest in healthy food prepared mainly at home with ingredients from local producers [57]. The impact of the COVID-19 pandemic on consumer behavior varies with countries, policies, and the spread of the infection [58]. For example, in Colombia, Brazil, and Chile, there was an increase in milk demand as producers in the UK, Canada, USA, and China were forced to dispose of significant amounts of milk due to a lack of consumer demand for the product [59,60]. Italian consumers began to consume more home-made foods, vegetables, and white meat [61]. In Great Britain, consumers indicated a conscious choice of a healthy lifestyle and limitation of unhealthy habits [62]. In turn, Polish consumers changed their food consumption patterns during the quarantine, eating more snacks, meat and dairy products, and fewer vegetables and fruits than before the pandemic [63]. Russian consumers, on the other hand, reduced the frequency of purchases and the number of products they bought but adopted healthier consumption patterns, consuming more fruit and vegetables and fewer snacks and pastries during the COVID-19 pandemic [64,65]. Interestingly, Burlea-Schiopoiu et al. noted that the COVID-19 crisis had a positive impact on food waste behavior among young consumers. Routine shopping, reusing leftovers, meal planning, and home cooking have become major behavioral control factors before the compulsive and obsessive buying process [66]. The consumption of organic fruit and vegetables and other health foods has also increased in response to higher caloric intake during the lockdown period and as a concern to increase and maintain resistance to the spread of the virus. The COVID-19 pandemic had a significant impact on consumers’ eating habits in terms of food preferences and food purchasing decisions [58].

Based on the above conditions, the purpose of our research is to analyze the shopping behavior of international students (Asian vs European) studying in Poland. This is the second part of our research; the first was on the tourism behavior of international students [67].

## 2. Materials and Methods

### 2.1. Ethical Approval

Approval for this study was obtained from the Ethics Committee of the University of Social Sciences (Warsaw, Poland)—resolution No. 01/2020 on the date 23 March 2020. All of the participants provided their informed consent before participating in the research.

### 2.2. Design of Questionnaire

The questionnaire consists of two parts. The first part consists of 20 items that cover the possible impacts of COVID-19 on the respondents’ tourism choices. The second part of the questionnaire (10 items) concerned the consumers’ purchasing behavior during the COVID-19 pandemic. The results of the analysis of 20 items concerning tourism choices were published in the article “How the COVID-19 pandemic influences the tourism behavior of the international students in Poland?” [67].

A 5-point rating scale was used, in which 5 = agree, 4 = somewhat agree, 3 = neither agree nor disagree, 2 = somewhat disagree, and 1 = disagree. The respondents were asked to rate the level from agreement to disagreement according to their judgment. The second part contained questions about age, working shifts, gender, financial situation, and region.

### 2.3. Study Design and Sample

The participants were recruited from universities located in Warsaw, Poland. The criteria for participation in the study were being a student and studying at a Polish university for a minimum of one year. After gaining approval from the Ethics Committee of the University of Social Sciences, the participants were invited to take part in the study. Due to the period of the study (COVID-19 pandemic), it was decided to choose the CAWI method (computer-assisted web interviewing technique). The survey was created in Google Forms, an online survey collection tool. The questionnaire was distributed to the students via MS Teams. All of the students used this program during distance learning.

A survey was carried out from June to September 2020. We reached out to over 3000 students and received 807 responses. Due to missing responses, 87 questionnaires were eliminated. A sample of 719 respondents was qualified for the final analysis. The characteristics of the study sample, considering socio-demographic features, are presented in Table 1.

Students aged 18 to 35 years and older participated in the survey. Over half of the respondents declared the age from 18 to 26. The least number of respondents were between 27 to 34 years old. 30.6% were aged 35 and above. Men slightly predominated in the study (54.0%). A total of 63.3% declared that they combined work and study. Less than half of the respondents declared that ‘I live sparingly and have enough money for my basic needs’. More than one-third of the respondents agreed with the statement ‘I have enough money for everything without special savings’. Only 4.3% described their situation as ‘I do not have enough money for my basic needs (such as food and clothes)’. Over half of the respondents declared Asian nationality (51.3%). More than one-third were from European countries (39.8%).

### 2.4. Data Analysis

Using descriptive analysis, the means, standard deviations (SD), minima, maxima, and frequencies (%) were calculated. The reliability of the scales was assessed using Cronbach’s alpha coefficient. In general, a value greater than 0.7 indicates satisfactory reliability [68]. Among other things, a low alpha value may be due to a small number of questions or weak interrelationships between the items. The distributions of the analyzed variables were checked by the Shapiro–Wilk test.

In the first step, an exploratory factor analysis (EFA) with varimax rotation was used to define the nature of the relationship between the factors. The number of factors was determined based on the following criteria: components with an eigenvalue of 1, a scree plot test, and the interpretability of the factors [69]. Information sources with factor loadings of at least 0.50 were considered. Data factorability was confirmed with the Kaiser–Meyer–Olkin (KMO) (cut-off value of 0.60) measure of sampling adequacy and Bartlett’s test of sphericity (*p* ≤ 0.05) [70].

The exploratory factor analysis was applied twice. First, the factor analysis was used considering the responses obtained from all respondents [N = 719]. Due to the small sample of respondents declaring a nationality other than European and Asian [N = 64], it was decided to conduct an exploratory factor analysis without the responses obtained from these respondents. Gorsuch [71] pointed to a sample size of 100 as the absolute minimum to conduct EFA, regardless of the number of items. Comrey and Lee [72] provided the sample size equal to 200 as fair to EFA. According to Lingard et al. [73], the use of factor analysis in small samples must be carefully considered and explicitly defended in terms of the ‘strength’ of the data. In both cases, the same criteria were adopted.

In the second step, we used multi-dimensional cluster analysis (CA). We used the partitioning method, which is to construct “k” data partitions from a database containing “n” objects. Each partition will represent a cluster, and k ≤ n. The clusters are formed by evaluating the similarities and dissimilarities of intrinsic characteristics between different cases. For each variable applied in our CA, we calculated the correlation ratio (CR) [74].

Cluster analysis was the last part of the analysis. We performed it using the k-means method as an algorithm that groups similar objects into groups called clusters. The result of cluster analysis is a set of clusters, where each cluster is distinct from every other cluster, and objects within each cluster are substantially similar to each other [75,76,77].

SPSS for Windows statistical software (8.0 version, IBM, Armonk, NY, USA) was used to perform statistical analyses.

## 3. Results

### 3.1. Purchasing Behaviors during the COVID-19 Pandemic

Most of the respondents agreed with the statement ‘My shopping behaviors have changed during COVID-19 period’ (mean 3.94) and ‘My shopping time is much shorter during the COVID-19 than before that period’ (Table 2). Certainly, such a high average response rate was influenced by the restrictions introduced in Poland during the pandemic (e.g., hours in stores only for seniors). Furthermore, fear of infection could have resulted in shorter time spent in the store. Many respondents agreed that they bought more fruits and vegetables during COVID-19 than before that period (mean of 3.91). This behavior may have been influenced by greater awareness of the impact of a healthy diet on health. However, on the other hand, some of the respondents declared that they bought more processed foods during COVID-19 than before that period (mean of 3.34). The respondents declared greater interest in shopping in smaller, local stores and greater interest in purchasing local products (‘I try to buy local products more often to support small businesses, especially in my area during COVID-19 period’—mean 3.81; ‘I shop more often in a local store than in supermarket during COVID-19 than before that period’—mean 3.50). Such behavior could have been caused by the desire to support local entrepreneurs. On the other hand, some respondents stated that during the COVID-19 pandemic, they were more likely to buy products online than in traditional stores (mean of 3.45). Some respondents said that also, after the COVID-19 pandemic, they will buy food more often online (mean of 3.29). Some people surveyed said they were buying more food than before the pandemic (I buy more products in store during COVID-19 than before that period—mean 3.48). The response to the statement ‘I spend more money on shopping during COVID-19 period’ is neutral with a mean of 3.08 (Table 2).

### 3.2. Explaratory Factor Analysis (EFA)

#### 3.2.1. EFA: All International Students

In Table 2, the primary variables have been presented. The respondents referred to them on a five-point scale. To examine the relationship between the observed variables with the use of a smaller number of unobserved variables, exploratory factor analysis was performed. The Kaiser–Meyer–Olkin value was 0.830. The obtained result indicated that the choice of analysis and the number of factors were correct. Bartlett’s test of sphericity *x*^2^ = 2143.293, *p* ≤ 0.01, indicated that correlations between items were high enough to perform the analysis.

The EFA was conducted using maximum likelihood extraction with varimax rotation (Table 3). The results of the EFA of the 10 items with a varimax rotation made it possible to extract two factors. Two factors were identified with an eigenvalue higher than the Kaiser criterion of 1. The first factor’s eigenvalue is 4.009, and it explains 40.09% of the variance. The second factor’s eigenvalue equals 1.404, which explains 14.04% of the variance. They explained 54.13% of the total variance. It has been arbitrarily assumed that the components of the factor are those variables that, after rounding, obtain absolute values equal to 0.5 or greater.

The Cronbach’s alpha coefficient of the total questionnaire (0.829) was within the recommended values. To assess the reliability of the applied tool, the Cronbach’s alpha coefficient was also calculated at the level of two dimensions. The results of the analysis indicate that it is possible to recognize the reliability of the research tool used (Table 3).

#### 3.2.2. EFA: Asian Students

EFA was performed considering the responses obtained from Asian students (Table 4).

The Kaiser–Meyer–Olkin value was 0.790. Bartlett’s test of sphericity *x*^2^ = 875.972, *p* ≤ 0.01. The conducted exploratory factor analysis allowed us to distinguish three factors. The EFA was conducted using maximum likelihood extraction with varimax rotation (Table 4). Three factors were identified with an eigenvalue higher than the Kaiser criterion of 1. The first factor’s eigenvalue is 3.761, and it explains 37.61% of the variance. The second factor’s eigenvalue equals 1.430, which explains 14.30% of the variance. The third factor’s eigenvalue is 1.080, which explains 10.80% of the variance. They explained 62.71% of the total variance. The components of the coefficient are those variables that, after rounding, obtain absolute values equal to or greater than 0.5.

#### 3.2.3. EFA: European Students

The next step was to carry out EFA into account the responses obtained from European students (Table 5). One item is not loaded under meaningful factors (‘I shop more often in a local store than in supermarket during COVID-19 than before that period’). Due to this fact, it was decided to exclude this item from the analysis. The analysis was performed for nine items.

The Kaiser–Meyer–Olkin value was 0.753. Bartlett’s test of sphericity *x*^2^ = 975.972, *p* ≤ 0.01. The conducted exploratory factor analysis allowed us to distinguish three factors. The EFA was conducted using maximum likelihood extraction with varimax rotation. Three factors were identified with an eigenvalue higher than the Kaiser criterion of 1. The first factor’s eigenvalue is 3.750, and it explains 37.50% of the variance. The second factor’s eigenvalue equals 1.381, which explains 13.82% of the variance. The third factor’s eigenvalue is 1.088, which explains 10.88% of the variance. They explained 62.19% of the total variance. The components of the coefficient are those variables that, after rounding, obtain absolute values equal to or greater than 0.5.

### 3.3. Cluster Analysis

#### 3.3.1. Cluster Analysis: All International Students

**Figure 1:**
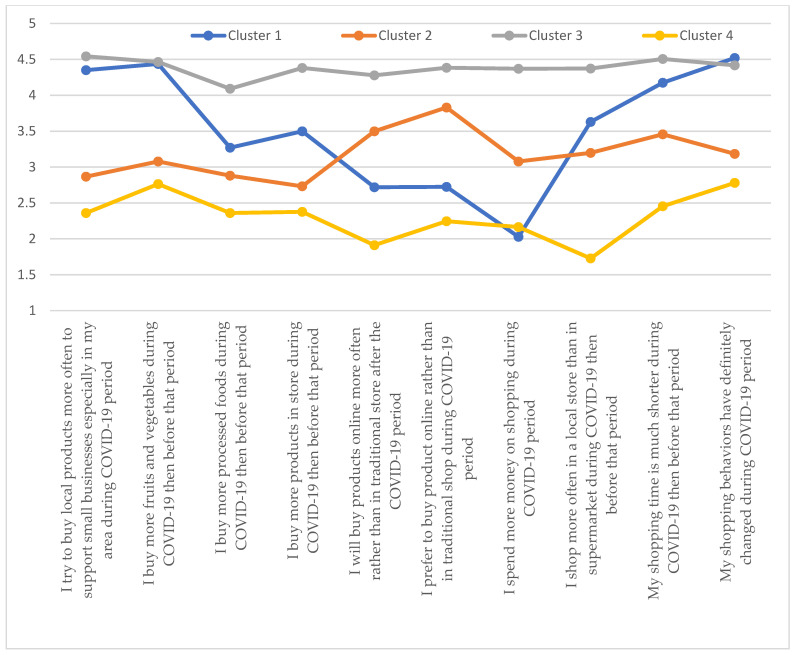
Cluster analysis: all international students.

For the whole surveyed population of international students studying in Warsaw, 4 clusters representing from 15.86% to 35.05% of the surveyed population were identified (Table 6). Cluster 3, representing 35.05% of the surveyed population, had the highest mean values for 8 of the 10 surveyed statements. The exception was the statement on the definitive change in shopping behavior and increased purchases of fruits and vegetables (a higher or comparable average value for cluster 1). Cluster 4 had the lowest mean values (the exception being the statement about spending more money during the COVID-19 pandemic). The largest spreads in average values were recorded for cluster 1, representing 29.4% of all international students. They ranged from 2.03 for the statement on spending more money during the COVID-19 pandemic to 4.52 for the statement on definitely changing shopping habits (Figure 1).

#### 3.3.2. Cluster Analysis: European Students

**Figure 2:**
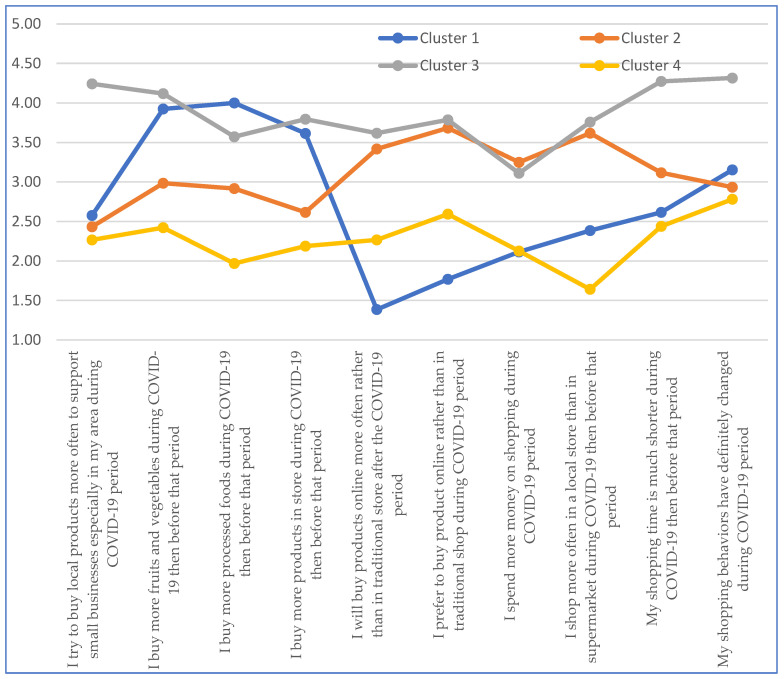
Cluster analysis: European students.

Among European students, four clusters were identified, representing 9.09% (cluster 1) to 47.55% (cluster 3) of the study population (Table 7). In cluster 3, the highest average value was obtained for 8 of the 10 statements studied. The exceptions were the statements relating to increased purchases of processed products and spending more money for purchases. The largest differences in mean values between cluster 3 and the other clusters were recorded for statement No. 1 (I try to buy local products more often to support small businesses especially in my area during COVID-19 period) and statement No. 10 (My shopping behaviors have definitely changed during COVID-19 period). Cluster No. 4, representing 22.38% of European students, had the lowest average values for 7 of the 10 statements studied. The exception was the statements on online shopping; here, the lowest averages were recorded for cluster 1, representing 9.09% of European students. In the case of the statement on increased spending, averages were obtained similar to those in cluster 1. In addition, cluster 1 had the greatest variation in the averages obtained, ranging from 1.38 for the statement I will buy products online more often rather than in traditional stores after the COVID-19 period to 4.00 for the statement I buy more processed foods during COVID-19 than before that period (Figure 2).

#### 3.3.3. Cluster Analysis: Asian Students

**Figure 3:**
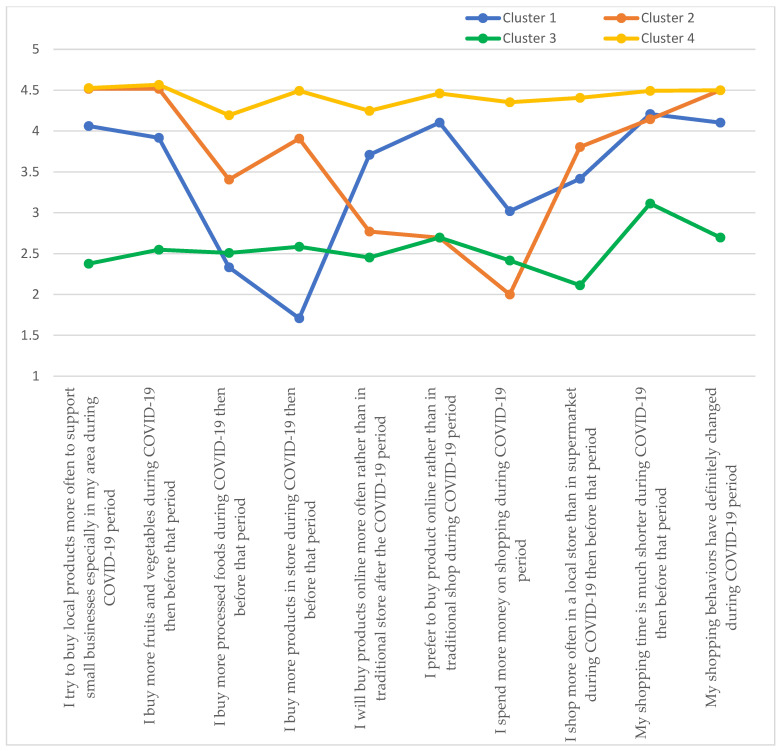
Cluster analysis: Asian students.

Among Asian students, there were four clusters representing 18.43% (cluster 1) to 34.42% (cluster 3) of the surveyed population of students from Asian countries (Table 8). Cluster 4, representing 27.37% of Asian students, had the highest averages for 7 of the 10 statements. The exceptions were statements related to buying more local produce and fruits and vegetables, as well as a definite change in shopping behavior during the COVID-19 pandemic. In these cases, the average values were obtained comparable to cluster 2, representing 18.43% of Asian students. For cluster 3, the lowest mean values were obtained for 6 of the 10 statements studied. The exceptions were statements related to buying more processed products, preferring online shopping, spending more money, and making larger purchases. Clusters 1 and 2 were characterized by significant variations in average values for the statements studied (Figure 3).

## 4. Discussion

The purpose of this paper was to analyze the shopping behavior of international students (European and Asian) during the COVID-19 pandemic. The paper performed factor analysis and cluster analysis for the entire population and separately for European and Asian students. The results obtained indicate a diversity of shopping behavior, which was confirmed by both factor and cluster analysis.

The mean values (on a five-point Likert scale) for the surveyed statements in Asian students’ responses were in the higher ranges, ranging from 3.15 for the statement about spending more money during COVID-19 to 4.19 for the statement about changing purchases during COVID-19. For students from Europe, averages ranged from 2.83 for the statement about buying more often from local stores than supermarkets during COVID-19 to 3.58 for the statement about preferring to buy local products to support local small businesses.

For students from Europe, averages ranged from 2.83 for the statement about buying more often from local stores than supermarkets during COVID-19 to 3.58 for the statement about preferring to buy local products to support local small businesses. Factor analysis for responses from European and Asian students indicated the presence of 3 factors, although the extent of the factors was different. The European students’ responses to the first factor included statements about a definite change in shopping behavior during COVID-19, buying local produce, buying more fruit, and shorter shopping time. Statements about buying more processed foods, shopping more, and spending more money were loaded into the second factor, while statements about online shopping during and after the pandemic were loaded into the third factor. In Asian students’ responses, the first factor included statements about definitely changing shopping behavior, buying local produce, in local stores buying more fruits and vegetables and spending less time shopping. The second factor referred to online shopping during and after the pandemic and devoting more time to shopping. The third factor referred to larger purchases, including processed foods.

Cluster analysis showed four clusters among Asian students and four clusters among European students. For Asian students, the distribution of cluster averages was in the higher values. The distribution of averages for cluster 4 ranged from 4.19 for the statement about buying more processed foods during COVID-19 to 4.57 for the statement about buying more fruits and vegetables and 4.53 for the statement about buying local produce more often. For European students, the highest average values were recorded for cluster 3, from 3.11 for the statement about spending more money on shopping during COVID-19 to 4.23 for the statement about definitely changing shopping behaviors during COVID-19 period.

The identified differences in the shopping behavior of European and Asian students may be due to three reasons. First, the pandemic began to spread in Asia. Wuhan, the epicenter of the COVID-19 virus, is the largest city in central China and a major transportation, industrial and commercial hub, with the largest railroad station, airport, and deep-water port [78]. The city’s air transport and its wide international reach contributed to the accelerated spread of COVID-19 outside China to countries such as Singapore, Japan, and Thailand [79], followed by other Asian and European countries. Currently, the distribution of diseases is dynamic and depends on many factors, including ecological, genetic, and human factors. At the same time, the ease of travel and movement has reduced the geographic barrier to microorganisms and increased the possibility of spreading infectious diseases [80]. We believe that the fact that the pandemic spread just from Asia caused a greater change in purchasing behavior among Asian students compared to European students. This manifested itself in greater online shopping during and after the pandemic, local shopping, and greater purchases of fruits and vegetables. Secondly, cultural differences are determined by the behavior of European and Asian consumers. The cultural approach indicates that Asian consumers are characterized by higher collectivist (vs. individualist) orientation, interdependent (vs. independent) self-construal and holistic (vs. analytic) thinking, and higher prevention (vs. promotion) orientation. In addition to the cultural syndromes, temporal orientation seems to be another important dimension in which Asians differ from Westerners. Asian consumers rely more on past events (versus present or future) in their decision-making process [81]. However, it is believed that one of the most distinctive characteristics of Asian customers is that they have a collectivist mentality [82]. In the literature, there are cross-cultural studies of consumer perception of brands, including luxury product brands [83], brand positioning using advertising [84], advertising perception [85], also restaurant atmosphere [86]. Other studies deal with the acceptance of Asian products and brands by European consumers [87], Western brands by Chinese consumers [88], comparison of the impact of shopping prescription consciousness on consumer decision-making styles [89], also Asian brand creation [90]. These differences are not due to an attitude of cultural ethnocentrism [91] but are the result of the differences of the cultural differences mentioned above. There are also studies of consumers from other cultures that indicate that culture is the principal explanation of consumer behavior disparities across countries, and so research on the impact of globalization on culture is essential. For example, comparing Chileans and Canadians, it has been proven that the strength of national identity and acculturation to global consumer culture play a role in consumer behavior [92].

Third, shopping attitudes and consumer optimism are different for Asian and European consumers. A study by research agency Millward Brown found that the feeling that shopping is an enjoyable experience, rather than an unpleasant obligation, is much stronger among Chinese than American consumers. In addition, 68% of Chinese respondents said they were “satisfied” with their shopping experience, compared to just 48% of American respondents. Chinese consumers are also more engaged than those in the U.S. in learning more about the products they are interested in buying. However, this is subject to change as people in the West are more concerned about their consumption habits, especially after the COVID-19 outbreak [93]. This may be because Asian consumers are more likely to seek help from others and mitigate minor conflicts. In addition, South Korean or Thai consumers would like, comment on or share on social media brands with which they emotionally identify or brands that would enhance their presentation [82].

The different expectations of Chinese and European consumers also apply to online shopping. Chinese customers have been found to have different expectations, including online chat support, product and service reviews, standard delivery within 1–2 days, and a full spectrum of payment methods [83]. This may be due to their consumer optimism. A McKinsey study found that most Asian consumers look to the future with more optimism than consumers from Europe or the United States. In addition, respondents from India and Indonesia are particularly optimistic about their countries’ economic recovery [94].

In conclusion, our study indicates the impact of the pandemic on the shopping behavior of European and Asian international students studying in Poland. As we wrote in the first part of our research, which has already been published, our study has some limitations. These are related to the timing of the survey, namely during the main wave of the pandemic, which translated into how we reached tourists. We did not reach representatives of students of all nationalities who studied in Warsaw, the capital of Poland. We did not survey international students in other Polish cities. As an explanation, let us use our assumption that Warsaw is the largest academic city in Poland in terms of the number of international students.

## 5. Conclusions

Our research has shown that purchasing behavior changed during the pandemic and according to statements, these changes will continue even after the pandemic ends. However, the response to the COVID-19 pandemic among Asian students was greater than among European students. This conclusion from our study should be subject to further research to determine the impact on future consumer behavior.

Referring to the managerial implications, entrepreneurs should apply new knowledge about consumer purchasing behavior and their changing needs. Firms should consider implementing new business models to cope with the rise in online shopping and the associated changes in consumer behavior. Ensuring fast delivery, the availability of various forms of payment, or ensuring the efficient handling of notifications/complaints submitted by customers certainly positively affects the company’s image in the eyes of consumers. During the pandemic, consumers spent less time shopping. This may translate into increased interest in shopping in smaller/local stores.

In addition to the management implementations mentioned, our research has other, broader practical significance. Since they apply to European and Asian students studying in Poland, the results of our study should be used by universities hosting foreign students, especially those as culturally diverse as the respondents in our survey. This will make it possible to adjust the real living conditions of international students to their cultural diversity; on the one hand, and on the other hand, it will contribute to raising awareness of cultural differences. In particular, the issue of increasing awareness of the existence of cultural differences manifested in all aspects of daily life, including shopping habits, will help level out possible misunderstandings and contribute to the acceptance and mutual enrichment of cultural values. Conducting education and awareness-raising campaigns will make it clear that cultural differences are not only differences at the level of values, religion, and customs but also that they are differences manifested in everyday life, including, for example, shopping. The theoretical implications arising from our study are equally significant. The results of our study—previous ones already published have dealt with tourist behavior—are a contribution to the identification of areas in which cultural differences manifest themselves. We believe that learning about all such areas will be beneficial for social welfare, mutual understanding, and tolerance.

Further research should pay attention to further developments in online commerce, lifestyle changes, and preferences for certain food products, including those of a dietary nature. This is especially important regarding young people, including students traveling in different parts of the world and gaining knowledge and work experience there. Further research should also be conducted in the exploration of cross-cultural differences and the areas in which these differences are expressed.

## Figures and Tables

**Table 1:** Characteristics of the studied group, considering selected socio-demographic characteristics.

Variables	Total	
	N = 719	(%)
Gender		
Female	331	46.0
Male	388	54.0
Age		
18–26	393	54.7
27–34	106	14.7
35 and above	220	30.6
Working shifts		
No	264	36.7
Yes	455	63.3
Financial situation		
I have enough money for everything without special savings	248	34.5
I live sparingly and have enough money for my basic needs	333	46.3
I live very sparingly to put aside money for my secondary needs	107	14.9
I do not have enough money for my basic needs (such as food and clothes)	31	4.3
Region		
Europe	286	39.8
Asia	369	51.3
Other	64	8.9

**Table 2:** Summary of the responses of the survey (N = 719).

Items	Mean *; SD	Median (Minimum–Maximum)
My shopping behaviors have definitely changed during COVID-19 period	3.94 ± 1.180	4 ** (1–5)
I buy more fruits and vegetables during COVID-19 than before that period	3.91 ± 1.155	4 ** (1–5)
My shopping time is much shorter during COVID-19 than before that period	3.88 ± 1.226	4 ** (1–5)
I try to buy local products more often to support small businesses especially in my area during COVID-19 period	3.81 ± 1.201	4 ** (1–5)
I shop more often in a local store than in supermarket during COVID-19 than before that period	3.50 ± 1.287	4 ** (1–5)
I buy more products in store during COVID-19 than before that period	3.48 ± 1.332	4 ** (1–5)
I prefer to buy product online rather than in traditional shop during COVID-19 period	3.45 ± 1.316	4 ** (1–5)
I buy more processed foods during COVID-19 than before that period	3.34 ± 1.260	3 ** (1–5)
I will buy products online more often rather than in traditional store after the COVID-19 period	3.29 ± 1.286	3 ** (1–5)
I spend more money on shopping during COVID-19 period	3.08 ± 1.442	3 ** (1–5)

* a five-point of scale—a rating of ‘1’ (disagree) means; a rating of ‘5’ (agree) means. ** distribution different than normal (Shapiro-Wilk test—*p* ≤ 0.05).

**Table 3:** The results of EFA.

Items	Factor 1	Factor 2	Cronbach’s Alpha	Total Cronbach’s Alpha
My shopping behaviors have definitely changed during COVID-19 period	0.723		0.802	0.829
I try to buy local products more often to support small businesses especially in my area during COVID-19 period	0.806			
I buy more fruits and vegetables during COVID-19 than before that period	0.772			
I buy more products in store during COVID-19 than before that period	0.507			
I shop more often in a local store than in supermarket during COVID-19 than before that period	0.563			
My shopping time is much shorter during COVID-19 than before that period	0.618			
I buy more processed foods during COVID-19 than before that period		0.516	0.716	
I prefer to buy product online rather than in traditional shop during COVID-19 period		0.800		
I will buy products online more often rather than in traditional store after the COVID-19 period		0.838		
I spend more money on shopping during COVID-19 period		0.655		
Variance explained (%)	40.09%	14.04%		
Total variance explained (%)	54.13%			

**Table 4:** The results of EFA: Asian students.

Items	Factor 1	Factor 2	Factor 3	Total Cronbach’s Alpha
My shopping behaviors have definitely changed during COVID-19 period	0.635			0.810
I try to buy local products more often to support small businesses especially in my area during COVID-19 period	0.806			
I buy more fruits and vegetables during COVID-19 than before that period	0.642			
I shop more often in a local store than in supermarket during COVID-19 than before that period	0.580			
My shopping time is much shorter during COVID-19 than before that period	0.690			
I prefer to buy product online rather than in traditional shop during COVID-19 period		0.870		
I will buy products online more often rather than in traditional store after the COVID-19 period		0.814		
I spend more money on shopping during COVID-19 period		0.552		
I buy more processed foods during COVID-19 than before that period			0.764	
I buy more products in store during COVID-19 than before that period			0.796	
Variance explained (%)	37.61%	14.30%	10.80%	
Total variance explained (%)	62.71%			

**Table 5:** The results of EFA: European students.

Items	Factor 1	Factor 2	Factor 3	Total Cronbach’s Alpha
My shopping behaviors have definitely changed during COVID-19 period	0.804			0.810
I try to buy local products more often to support small businesses especially in my area during COVID-19 period	0.752			
I buy more fruits and vegetables during COVID-19 than before that period	0.612			
My shopping time is much shorter during COVID-19 than before that period	0.675			
I buy more processed foods during COVID-19 than before that period		0.641		
I buy more products in store during COVID-19 than before that period		0.710		
I spend more money on shopping during COVID-19 period		0.737		
I prefer to buy product online rather than in traditional shop during COVID-19 period			0.838	
I will buy products online more often rather than in traditional store after the COVID-19 period			0.834	
Variance explained (%)	37.50%	13.82%	10.88%	
Total variance explained (%)	62.19%			

**Table 6:** Cluster analysis: all international students.

Specification	All Students	Cluster 1	Cluster 2	Cluster 3	Cluster 4	
Number of students	719	211	142	252	114	
Number of students (%)	100%	29.35%	19.75%	35.05%	15.86%	
I try to buy local products more often to support small businesses especially in my area during COVID-19 period	3.81	4.35	2.87	4.54	2.36	*p* < 0.001
I buy more fruits and vegetables during COVID-19 than before that period	3.91	4.44	3.08	4.46	2.76	*p* < 0.001
I buy more processed foods during COVID-19 than before that period	3.34	3.27	2.88	4.09	2.36	*p* < 0.001
I buy more products in store during COVID-19 than before that period	3.48	3.50	2.73	4.38	2.38	*p* < 0.001
I will buy products online more often rather than in traditional store after the COVID-19 period	3.29	2.72	3.50	4.28	1.91	*p* < 0.001
I prefer to buy product online rather than in traditional shop during COVID-19 period	3.45	2.73	3.83	4.38	2.25	*p* < 0.001
I spend more money on shopping during COVID-19 period	3.08	2.03	3.08	4.37	2.17	*p* < 0.001
I shop more often in a local store than in supermarket during COVID-19 than before that period	3.50	3.63	3.20	4.37	1.73	*p* < 0.001
My shopping time is much shorter during COVID-19 than before that period	3.88	4.18	3.46	4.51	2.46	*p* < 0.001
My shopping behaviors have definitely changed during COVID-19 period	3.94	4.52	3.18	4.42	2.78	*p* < 0.001

**Table 7:** Cluster analysis: European students.

Specification	European Students	Cluster 1	Cluster 2	Cluster 3	Cluster 4	
Number of students	286	26	60	136	64	
Number of students (%)	100%	9.09%	20.98%	47.55%	22.38%	
I try to buy local products more often to support small businesses especially in my area during COVID-19 period	3.58	2.58	2.43	4.24	2.27	*p* < 0.001
I buy more fruits and vegetables during COVID-19 than before that period	3.27	3.92	2.98	4.12	2.42	*p* < 0.001
I buy more processed foods during COVID-19 than before that period	3.48	4.00	2.92	3.57	1.97	*p* < 0.001
I buy more products in store during COVID-19 than before that period	3.12	3.62	2.62	3.79	2.19	*p* < 0.001
I will buy products online more often rather than in traditional store after the COVID-19 period	3.17	1.38	3.42	3.62	2.27	*p* < 0.001
I prefer to buy product online rather than in traditional shop during COVID-19 period	3.31	1.77	3.68	3.79	2.59	*p* < 0.001
I spend more money on shopping during COVID-19 period	3.07	2.12	3.25	3.11	2.13	*p* < 0.001
I shop more often in a local store than in supermarket during COVID-19 than before that period	2.83	2.38	3.62	3.76	1.64	*p* < 0.001
My shopping time is much shorter during COVID-19 than before that period	3.13	2.62	3.12	4.27	2.44	*p* < 0.001
My shopping behaviors have definitely changed during COVID-19 period	3.47	3.15	2.93	4.32	2.78	*p* < 0.001

**Table 8:** Cluster analysis: Asian students.

Specification	Asian Students	Cluster 1	Cluster 2	Cluster 3	Cluster 4	
Number of students	369	68	73	127	101	
Number of students (%)	100%	18.43%	19.78%	34.42%	27.37%	
I try to buy local products more often to support small businesses especially in my area during COVID-19 period	4.15	4.06	4.52	2.38	4.53	*p* < 0.001
I buy more fruits and vegetables during COVID-19 than before that period	4.18	3.92	4.52	2.55	4.57	*p* < 0.001
I buy more processed foods during COVID-19 than before that period	3.46	2.33	3.41	2.51	4.19	*p* < 0.001
I buy more products in store during COVID-19 than before that period	3.67	1.71	3.91	2.58	4.49	*p* < 0.001
I will buy products online more often rather than in traditional store after the COVID-19 period	3.45	3.71	2.77	2.45	4.25	*p* < 0.001
I prefer to buy product online rather than in traditional shop during COVID-19 period	3.60	4.10	2.69	2.70	4.46	*p* < 0.001
I spend more money on shopping during COVID-19 period	3.15	3.02	2.00	2.42	4.35	*p* < 0.001
I shop more often in a local store than in supermarket during COVID-19 than before that period	3.76	3.42	3.81	2.11	4.41	*p* < 0.001
My shopping time is much shorter during COVID-19 than before that period	4.15	4.21	4.14	3.11	4.49	*p* < 0.001
My shopping behaviors have definitely changed during COVID-19 period	4.19	4.10	4.50	2.70	4.50	*p* < 0.001

## Data Availability

Data are available at the Department of Food Market and Consumption research in the Institute of Human Nutrition Sciences, Warsaw University of Life Sciences, in Poland.

## References

[1] Bawa K., Ghosh A. (1999). A Model of Household Grocery Behavior Shopping. Mark. Lett..

[2] Wang Y., Xu R., Schwartz M., Ghosh D., Chen X. (2020). COVID-19 and Retail Grocery Management: Insights from a Broad-Based Consumer Survey. IEEE Eng. Manag. Rev..

[3] Cici E.N., Bilginer Özsaatci F.G. (2021). The Impact of Crisis Perception on Consumer Purchasing Behaviors during the COVID-19 (Coronavirus) Period: A Research on Consumers in Turkey. Eskişeh. Osman. Üniv. İktis. İdari Bilim. Derg..

[4] Rozin P. (1988). Cultural Approaches to Human Food Preferences. Nutr. Modul. Neural Funct..

[5] Sulmont-Rossé C., Drabek R., Almli V.L., van Zyl H., Silva A.P., Kern M., McEwan J.A., Ares G. (2019). A cross-cultural perspective on feeling good in the context of foods and beverages. Food Res. Int..

[6] Doole I., Lowe R. (2008). International Marketing Strategy: Analysis, Development and Implementation.

[7] Laaksonen O., Ma X., Pasanen E., Zhou P., Yang B., Linderborg K.M. (2020). Sensory Characteristics Contributing to Pleasantness of Oat Product Concepts by Finnish and Chinese Consumers. Foods.

[8] Choi J.H., Gwak M.J., Chung S.J., Kim K.O., O’Mahony M., Ishii R., Bae Y.W. (2015). Identifying the drivers of liking by investigating the reasons for (dis)liking using CATA in cross-cultural context: A case study on barbecue sauce. J. Sci. Food Agric..

[9] Hay C., de Matos A.D., Low J., Feng J., Lu D., Day L., Hort J. (2021). Comparing cross-cultural differences in perception of drinkable yoghurt by Chinese and New Zealand European consumers. Int. Dairy J..

[10] De Mooij M., Hofstede G. (2011). Cross-cultural consumer behavior: A review of research findings. J. Int. Consum. Mark..

[11] Kire K., Rajkumar P. (2017). Culture Influence on Consumer Behavior. IJARIIE.

[12] Limayem M., Khalifa M., Frini A. (2000). What makes consumers buy from Internet? A longitudinal study of online shopping. IEEE Trans. Syst. Man Cybern. Part A Systems Humans.

[13] Mahmood M.A., Bagchi K., Ford T.C. (2004). On-line shopping behavior: Cross-country empirical research. Int. J. Electron. Commer..

[14] Young C.A., Corsun D.L., Baloglu S. (2007). A taxonomy of hosts visiting friends and relatives. Ann. Tour. Res..

[15] Senecal S., Kalczynski P.J., Nantel J. (2005). Consumers’ decision-making process and their online shopping behavior: A clickstream analysis. J. Bus. Res..

[16] Payne J.W., Bettman J.R., Johnson E. (1993). The Adaptive Decision Maker.

[17] Loketkrawee P., Bhatiasevi V. (2018). Elucidating the Behavior of Consumers toward Online Grocery Shopping: The Role of Shopping Orientation. J. Internet Commer..

[18] Ingene C.A. (1984). Temporal influences upon spatial shopping behavior of consumers. Pap. Reg. Sci. Assoc..

[19] Gupta A., Su B.C., Walter Z. (2004). Risk profile and consumer shopping behavior in electronic and traditional channels. Decis. Support Syst..

[20] Alba J., Lynch J., Weitz B., Janiszewski C., Lutz R., Wood S., Alba J., Lynch J., Weitz B., Janiszewski C. (2013). Interactive Home Shopping: Consumer, Retailer, and Manufacturer Incentives to Participate in Electronic Marketplaces. J. Mark..

[21] Yin S. (2022). Psychological Analysis on the Consumers’ Online Shopping Behavior. Proceedings of the 2022 International Conference on Social Sciences and Humanities and Arts (SSHA 2022).

[22] Rosen K.T., Howard A.L. (2000). E-Retail: Gold Rush or Fool’s Gold?. Calif. Manag. Rev..

[23] King M.F., Balasubramanian S.K. (1994). The effects of expertise, end goal, and product type on adoption of preference formation strategy. J. Acad. Mark. Sci..

[24] Bearden W.O., Etzel M.J. (1982). Reference Group Influence on Product and Brand Purchase Decisions. J. Consum. Res..

[25] Childers T.L., Rao A.R. (1992). The Influence of Familial and Peer-based Reference Groups on Consumer Decisions. J. Consum. Res..

[26] Bakos J.Y. (1997). Reducing Buyer Search Costs: Implications for Electronic Marketplaces. Manag. Sci..

[27] Perea Y., Monsuwé T., Dellaert B.G.C., De Ruyter K. (2004). What drives consumers to shop online? A literature review. Int. J. Serv. Ind. Manag..

[28] Lakshmanan A. (2016). Correlations of Biomechanical Characteristics with Ball Speed in Penalty Corner Push-In Customers Satisfaction towards Online Shopping in Amazon.Com-A Study with Reference to Udumalpet Taluk. Int. J. Recent Res. Appl. Stud..

[29] Evanschitzky H., Iyer G.R., Hesse J., Ahlert D. (2004). E-satisfaction: A re-examination. J. Retail..

[30] Szymanski D.M., Hise R.T. (2000). E-satisfaction: An initial examination. J. Retail..

[31] Juniwati J. (2014). Influence of Perceived Usefulness, Ease of Use, Risk on Attitude and Intention to Shop Online. Eur. J. Bus. Manag..

[32] Taylor J.W. (2018). The Role of Risk in Consumer Behavior: A comprehensive and operational theory of risk taking in consumer behavior. J. Mark..

[33] Freudenburg W.R. (1988). Perceived Risk, Real Risk: Social Science and the Art of Probabilistic Risk Assessment. Science.

[34] Holguín-Veras J., Taniguchi E., Jaller M., Aros-Vera F., Ferreira F., Thompson R.G. (2014). The Tohoku disasters: Chief lessons concerning the post disaster humanitarian logistics response and policy implications. Transp. Res. Part A Policy Pract..

[35] Sweeney J.C., Soutar G.N., Johnson L.W. (1999). The role of perceived risk in the quality-value relationship: A study in a retail environment. J. Retail..

[36] Gonzalez-Feliu J., Chong M., Vargas-Florez J., de Brito I., Osorio-Ramirez C., Piatyszek E., Altamirano R.Q. (2020). The maturity of humanitarian logistics against recurrent crises. Soc. Sci..

[37] Rossolov A., Aloshynskyi Y., Lobashov O. (2022). How COVID-19 Has Influenced the Purchase Patterns of Young Adults in Developed and Developing Economies: Factor Analysis of Shopping Behavior Roots. Sustainability.

[38] Ducret R. (2014). Parcel deliveries and urban logistics: Changes and challenges in the courier express and parcel sector in Europe—The French case. Res. Transp. Bus. Manag..

[39] Hagen T., Scheel-Kopeinig S. (2021). Would customers be willing to use an alternative (chargeable) delivery concept for the last mile?. Res. Transp. Bus. Manag..

[40] Kaytaz M., Gul M.C. (2014). Consumer response to economic crisis and lessons for marketers: The Turkish experience. J. Bus. Res..

[41] Baker S.R., Farrokhnia R.A., Meyer S., Pagel M., Yannelis C. (2020). How does household spending respond to an epidemic? consumption during the 2020 COVID-19 pandemic. Rev. Asset Pricing Stud..

[42] Piyapromdee S., Spittal P. (2020). The Income and Consumption Effects of COVID-19 and the Role of Public Policy. Fisc. Stud..

[43] Gu S., Ślusarczyk B., Hajizada S., Kovalyova I., Sakhbieva A. (2021). Impact of the COVID-19 pandemic on online consumer purchasing behavior. J. Theor. Appl. Electron. Commer. Res..

[44] Baarsma B., Groenewegen J. (2021). COVID-19 and the Demand for Online Grocery Shopping: Empirical Evidence from the Netherlands. De Economist.

[45] Chang H.H., Meyerhoefer C.D. (2021). COVID-19 and the Demand for Online Food Shopping Services: Empirical Evidence from Taiwan. Am. J. Agric. Econ..

[46] Timotius E., Octavius G.S. (2021). Global Changing of Consumer Behavior to Retail Distribution due to Pandemic of COVID-19: A Systematic Review. J. Distrib. Sci..

[47] Pantano E., Pizzi G., Scarpi D., Dennis C. (2020). Competing during a pandemic? Retailers’ ups and downs during the COVID-19 outbreak. J. Bus. Res..

[48] Grashuis J., Skevas T., Segovia M.S. (2020). Grocery shopping preferences during the COVID-19 pandemic. Sustainability.

[49] Abbu H.R., Fleischmann D., Gopalakrishna P. (2021). The Digital Transformation of the Grocery Business—Driven by Consumers, Powered by Technology, and Accelerated by the COVID-19 Pandemic. Adv. Intell. Syst. Comput..

[50] Wiranata A.T., Hananto A. (2020). Do Website Quality, Fashion Consciousness, and Sales Promotion Increase Impulse Buying Behavior of E-Commerce Buyers?. Indones. J. Bus. Entrep..

[51] Addo P.C., Jiaming F., Kulbo N.B., Liangqiang L. (2020). COVID-19: Fear appeal favoring purchase behavior towards personal protective equipment. Serv. Ind. J..

[52] Shamim K., Ahmad S., Alam M.A. (2021). COVID-19 health safety practices: Influence on grocery shopping behavior. J. Public Aff..

[53] PwC Evolving Priorities: COVID-19 Rapidly Reshapes Consumer Behavior. https://www.pwc.com/us/en/industries/consumer-markets/library/covid-19-consumer-behavior-survey.html.

[54] Islam T., Pitafi A.H., Arya V., Wang Y., Akhtar N., Mubarik S., Xiaobei L. (2020). Panic buying in the COVID-19 pandemic: A multi-country examination. J. Retail. Consum. Serv..

[55] Taha V.A., Pencarelli T., Škerháková V., Fedorko R., Košíková M. (2021). The Use of Social Media and Its Impact on Shopping Behavior of Slovak and Italian Consumers during COVID-19 Pandemic. Sustainability.

[56] Fanelli R.M. (2021). Changes in the Food-Related Behaviour of Italian Consumers during the COVID-19 Pandemic. Foods.

[57] Filimonau V., Vi L.H., Beer S., Ermolaev V.A. (2022). The COVID-19 pandemic and food consumption at home and away: An exploratory study of English households. Socio-Econ. Plan. Sci..

[58] Dumitras D.E., Harun R., Arion F.H., Chiciudean D.I., Kovacs E., Oroian C.F., Porutiu A., Muresan I.C. (2021). Food Consumption Patterns in Romania during the COVID-19 Pandemic. Foods.

[59] Acosta A., McCorriston S., Nicolli F., Venturelli E., Aratchilage U.G., ArceDiaz E., Scudiero L., Sammartino A., Schneider F., Steinfeld H. (2021). Immediate effects of COVID-19 on the global dairy sector. Agric. Syst..

[60] Weersink A., von Massow M., Bannon N., Ifft J., Maples J., McEwan K., McKendree M.G.S., Nicholson C., Novakovic A., Rangarajan A. (2021). COVID-19 and the agri-food system in the United States and Canada. Agric. Syst..

[61] Di Renzo L., Gualtieri P., Pivari F., Soldati L., Attinà A., Cinelli G., Cinelli G., Leggeri C., Caparello G., Barrea L. (2020). Eating habits and lifestyle changes during COVID-19 lockdown: An Italian survey. J. Transl. Med..

[62] Snuggs S., McGregor S. (2021). Food & meal decision making in lockdown: How and who has COVID-19 affected?. Food Qual. Prefer..

[63] Sidor A., Rzymski P. (2020). Dietary Choices and Habits during COVID-19 Lockdown: Experience from Poland. Nutrients.

[64] Ben Hassen T., El Bilali H., Allahyari M.S., Berjan S., Fotina O. (2021). Food purchase and eating behavior during the COVID-19 pandemic: A cross-sectional survey of Russian adults. Appetite.

[65] Reznik A., Gritsenko V., Konstantinov V., Khamenka N., Isralowitz R. (2021). COVID-19 Fear in Eastern Europe: Validation of the Fear of COVID-19 Scale. Int. J. Ment. Health Addict..

[66] Burlea-Schiopoiu A., Ogarca R.F., Barbu C.M., Craciun L., Baloi I.C., Mihai L.S. (2021). The impact of COVID-19 pandemic on food waste behaviour of young people. J. Clean. Prod..

[67] Szlachciuk J., Kulykovets O., D’Ebski M., Krawczyk A., Górska-Warsewicz H. (2022). How Has the COVID-19 Pandemic Influenced the Tourism Behaviour of International Students in Poland?. Sustainability.

[68] George D., Mallery P. (2003). SPSS for Windows Step by Step: A Simple Guide and Reference, 11.0 Update.

[69] Ledesma R.D., Valero-Mora P., Macbeth G. (2015). The Scree Test and the Number of Factors: A Dynamic Graphics Approach. Span. J. Psychol..

[70] Shkeer A.S., Awang Z. (2019). Exploring the items for measuring the marketing information system construct: An exploratory factor analysis. Int. Rev. Manag. Mark..

[71] Gorsuch R.L., Schinka J.A., Velicer W.F. (2003). Factor analysis. Handbook of Psychology: Research Methods in Psychology.

[72] Comrey A.L., Lee H.B. (2013). A First Course in Factor Analysis.

[73] Lingard H.C., Lingard H.C., Rowlinson S. (2006). Sample size in factor analysis: Why size matters. Retrieved Data.

[74] Tavakol M., Dennick R. (2011). Making sense of Cronbach’s alpha. Int. J. Med. Educ..

[75] Goel S., Bansal A., Sharma M., Pradesh U., Goel I.S. (2017). Improved K-mean Clustering Algorithm for Prediction Analysis using Classification Technique in Data Mining. Artic. Int. J. Comput. Appl..

[76] Ali A., Sheng-Chang C. (2020). Characterization of well logs using K-mean cluster analysis. J. Pet. Explor. Prod. Technol..

[77] Yadav J., Sharma M. (2013). A Review of K-mean Algorithm. Int. J. Eng. Trends Technol..

[78] Wilson M.E., Chen L.H. (2020). Travellers give wings to novel coronavirus (2019-nCoV). J. Travel Med..

[79] Bogoch I.I., Watts A., Thomas-Bachli A., Msa C.H., Dphil M.U.G.K., Khan K. (2020). Potential for global spread of a novel coronavirus from China. J. Travel Med..

[80] Mc Baker D.A. (2015). Tourism and the Health Effects of Infectious Diseases: Are There Potential Risks for Tourists?. Int. J. Saf. Secur. Tour./Hosp..

[81] Ha Y.-W. (2015). The Psychology of Asian Consumers: What We Know and What We Don’t. Acad. Asian Bus. Rev..

[82] The Difference Between Asian and Western Customers When Talking to Brands|by Robbie Mukai|Better Marketing. https://bettermarketing.pub/the-difference-between-asian-and-western-customers-when-talking-to-brands-999924f8c70e.

[83] How Chinese and European Consumer Expectations Differ. https://cosmeticsbusiness.com/news/article_page/How_Chinese_and_European_consumer_expectations_differ/125178.

[84] Alden D.L., Steenkamp J.B.E.M., Batra R. (2018). Brand Positioning through Advertising in Asia, North America, and Europe: The Role of Global Consumer Culture. J. Mark..

[85] Zhou N., Belk R.W. (2004). Chinese consumer readings of global and local advertising appeals. J. Advert..

[86] Kim K.-B., Kang S.-Y., Park S.-H. (2015). Perceiving the Atmosphere of Asian Restaurants: European Customers vs. Asian Customer. Int. J. Tour. Sci..

[87] Leonidou L.C., Hadjimarcou J., Kaleka A., Stamenova G.T. (1999). Bulgarian consumers’ perceptions of products made in Asia Pacific. Int. Mark. Rev..

[88] Long F., Bhuiyan M.A., Aziz N.A., Rahman M.K. (2022). Predicting young Chinese consumers’ intentions to purchase Western brands: Structural model analysis. PLoS ONE.

[89] Lamour C., De La Robertie C. (2016). Prescribed consumption and consumers’ decision-making styles: A cross-cultural comparison between Europe and Asia. Int. J. Retail Distrib. Manag..

[90] Cayla J., Eckhardt G.M. (2008). Asian brands and the shaping of a transnational imagined community. J. Consum. Res..

[91] Rašković M., Ding Z., Hirose M., Žabkar V., Fam K.S. (2020). Segmenting young-adult consumers in East Asia and Central and Eastern Europe—The role of consumer ethnocentrism and decision-making styles. J. Bus. Res..

[92] Cleveland M., Rojas-Méndez J.I., Laroche M., Papadopoulos N. (2016). Identity, culture, dispositions and behavior: A cross-national examination of globalization and culture change. J. Bus. Res..

[93] Comparing Chinese and Western Consumer Habits|What Marketers Should Know. https://daxueconsulting.com/western-consumer-habits/.

[94] Keeping Pace with Asian Consumer Trends|McKinsey. https://www.mckinsey.com/industries/retail/our-insights/catering-to-asian-consumers.

